# High-efficiency oxygen evolution electrocatalysis enabled by Ar/O_2_ plasma-induced synergistic modifications in NiFe Prussian blue analogue systems

**DOI:** 10.1039/d5ra03979g

**Published:** 2025-09-30

**Authors:** Yaoyao He, Jin Fang, Wenyuan Zhang, Nnditshedzeni Eric Maluta, Fhulufhelo Nemangwele, Li Zhang, Hui Lv, Pei Hu, Zhuo Peng

**Affiliations:** a China-South Africa PV-Hydrogen Energy Joint Research Center, School of Science, Hubei University of Technology (HBUT) Wuhan 430068 China 20211086@hbut.edu.cn; b Department of Physics, University of Venda Thohoyandou 0950 South Africa; c School of Physics and Electromechanical Engineering, Hubei University of Education Wuhan 430068 China

## Abstract

The advancement of efficient and durable non-precious-metal oxygen evolution reaction (OER) electrocatalysts is pivotal for progressing green hydrogen generation *via* water electrolysis. Traditional metal–organic frameworks (MOFs) are constrained by their low conductivity and structural degradation during pyrolysis, hindering electrocatalytic performance. In this study, we introduce a synergistic Ar/O_2_ plasma etching approach to reconfigure NiFe Prussian blue analogues (PBA) anchored on nickel foam (NF), simultaneously addressing structural and electronic limitations. The hybrid plasma treatment integrates physical Ar bombardment and chemical O_2_ oxidation, selectively eliminating C/N ligands to expose active catalytic interfaces while creating porosity (20–50 nm pores) and stabilizing high-spin Fe^3+^ species. Structural analyses (SEM, TEM, XRD) validate maintained framework integrity alongside optimized surface roughness and mesoporous networks. XPS investigations demonstrate plasma-induced electronic modulation, wherein O_2_ plasma oxidizes Fe^2+^ to Fe^3+^ and generates conductive Ni/Fe oxides, thereby refining intermediate adsorption/desorption kinetics. Electrochemical evaluations reveal superior OER activity: the NiFe PBA-Ar/O_2_ catalyst attains a low overpotential of 334 mV at 100 mA cm^−2^, a reduced Tafel slope of 97 mV dec^−1^, and remarkable operational stability (40 mV degradation over 20 h at 100 mA cm^−2^), exceeding the performance of both Ar-treated counterparts and pristine NiFe PBA. This work establishes a universal non-equilibrium plasma engineering strategy for designing MOF-derived electrocatalysts, resolving the trade-off between structural robustness and catalytic efficiency for sustainable energy applications.

## Abstract 2

Ar/O_2_-plasma reconstructed NiFe PBA on Ni foam: 334 mV to drive 100 mA cm^−2^ for durable OER.

## Graphical abstract

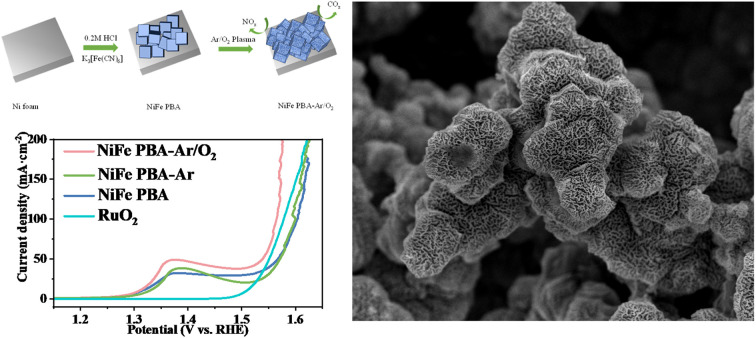

## Introduction

Against the background of the transition of global energy structure towards decarbonization and the realization of the goal of “carbon neutrality”, the development of highly efficient and scalable renewable energy technologies has become a central issue in the response to the climate crisis. The industrialization of water electrolysis as a key pathway for green hydrogen production is still limited by the high overpotential and kinetic hysteresis of the anodic oxygen extraction reaction (OER), which involves a four-electron transfer process (4OH^−^ → O_2_ + 2H_2_O + 4e^−^) with a significant increase in electrode polarization due to the energy barrier.^1–3^ The energy barrier leads to a significant increase in electrode polarization, which accounts for more than 90% of the overall energy consumption of the electrolytic water system. Although noble metal catalysts represented by IrO_2_/RuO_2_ can reduce the overpotential, their scarcity of resources and high cost severely restrict large-scale applications.^4–7^ Therefore, the development of non-precious-metal OER catalysts that combine high activity, high stability and low cost is the difficulty of current research.^8^ In recent years, metal–organic frameworks (MOFs) have become highly promising OER catalysts by virtue of their designable three-dimensional pore structure, atomically dispersed metal activity centers, and high specific surface area.^9–11^ However, conventional MOFs also face bottlenecks in electrocatalytic applications, such as poor intrinsic conductivity due to the insulating nature of their organic ligands, low electron transport efficiency, and pyrolysis treatment leading to pore collapse with active site agglomeration.^12–16^

In response to the above problems, non-equilibrium plasma technology has been applied to the functionalization modification of MOFs in recent years due to its unique advantage of realizing the material surface reconstruction under mild conditions (low temperature and atmospheric pressure).^17–19^ For example, high-energy Ar^+^ plasma bombardment selectively strips organic ligands from the surface of MOFs, exposing metal nodes in the subsurface layer and forming a multilevel pore structure to facilitate mass transfer with pore sizes up to 20–50 nm. However, there are functional limitations to single-gas etching: although pure Ar plasma can enhance the surface roughness, it lacks the ability to modulate the metal electronic state ability to solve the problem of insufficient endowment activity in the active sites;^20,21^ and although pure O_2_ plasma can improve electron transport by oxidizing to generate conductive metal oxides (*e.g.*, NiO_*x*_, FeO_*x*_), excessive oxidation destroys the periodicity of the skeleton of the MOFs (disappearance of the diffraction peaks of the crystalline surfaces in the XRD), which leads to the decrease of the mechanical strength.^22–24^

Inspired by reactive ion etching (RIE) in microelectronic fabrication, plasma source gas mixtures—by combining components with distinct functions—not only enhance chemical etching activity but also modulate physical bombardment intensity. The synergistic effects of these gas mixtures further maintain plasma stability and improve etching uniformity. Therefore, this work proposes to use Ar/O_2_ mixture for RIE of NiFe PBA, which utilizes the synergistic effect of Ar and O_2_ on NiFe PBA to reconfigure the oriented structure of the Prussian blue analogues, achieve the maximum exposure of the catalytically active interfaces, and effectively improve the catalytic activity of MOFs materials.^25–29^ This low-temperature and rapid hybrid gas plasma treatment method is expected to be extended to the surface modification of a wider range of MOF-based electrocatalysts.

## Results and discussion

**Fig. 1:**
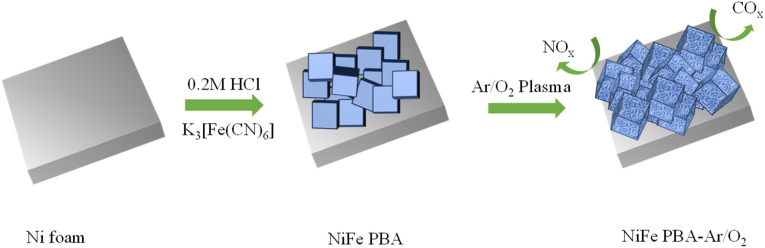
Catalyst preparation flowchart.

The synthesis of NiFe PBA-Ar/O_2_ composite is shown in Fig. 1. Nickel foam was used as a metal–organic support skeleton and as a source of Ni. In acidic environments, the ferricyanide ion ([Fe(CN)_6_]^3−^) accelerates the oxidation of metallic nickel (Ni^0^), generating nickel ions (Ni^2+^) and ferrocyanide ions ([Fe(CN)_6_]^4−^). This process ultimately yields a dual-phase product comprising nickel ferricyanide (Ni_3_[Fe(CN)_6_]_2_) and nickel ferrocyanide (Ni_2_[Fe(CN)_6_]). Finally, NiFe PBA-Ar/O_2_ was obtained by reactive ion etching using a gas mixture.

**Fig. 2:**
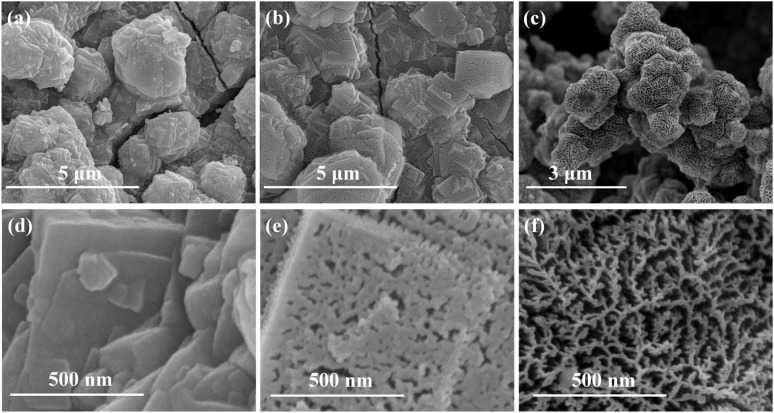
SEM photographs of catalysts NiFe PBA (a and d), NiFe PBA-Ar (b and e) and NiFe PBA-Ar/O_2_ (c and f).

Each of the prepared catalysts was characterized separately by scanning electron microscopy (SEM) and X-ray diffraction (XRD). The results are shown in Fig. 2. The microscopic morphology of the prepared NiFe Prussian blue analog (NiFe PBA), in (Fig. 2a), showed a polygonal stacked granular structure, which was different from that of the conventional Prussian blue analog with a cubic structure, but the diffraction peaks from XRD (Fig. 4a) were consistent with the standard card, and no other heterogeneous peaks were observed. This result indicates the successful synthesis of pure NiFe-PBA/NF precursor. The prepared NiFe PBA precursor was treated with Ar/O_2_ binary gas plasma to obtain NiFe PBA-Ar/O_2_. The resulting material was also characterized by XRD, and no obvious new XRD peaks were observed for NiFe PBA-Ar/O_2_., which suggests that the material retains the morphology of NiFe PBA precursor. NF precursor morphology and its metal–organic framework structure was retained after plasma treatment, which increased the specific surface area of the catalyst compared with the conventional NiFe-based oxygen precipitation electrocatalyst. By further magnifying the SEM image (Fig. 2d), it can be observed that the granular Prussian blue analogs are also characterized by flat ribs and their surfaces are approximately smooth.

The sample (NiFe PBA-Ar) after etching of NiFe PBA by passing Ar only was also characterized as shown in (Fig. 2b). It seems that there is no difference in appearance from (Fig. 2a), but from the magnified view (Fig. 2e), it was noted that many pores appeared on the surface. This structure is attributed to the high-energy argon ions (Ar^+^) in the argon plasma bombarding the NiFe PBA surface. It can be seen that some atoms or molecules on the surface are removed, increasing the surface roughness and exposing more active sites.^30^

Finally, NiFe PBA-Ar/O_2_. was characterized and the results are shown in (Fig. 2c). Compared to (Fig. 2a and b) some dense shadows appear on the surface, and by further magnification in (Fig. 2f) it can be observed that the pores on the surface of the sample become larger, with pore diameters in the order of tens of nanometers, as well as higher densities. As we know that O_2_ has high reactivity, compared with the etching by simply passing Ar, the introduction of O_2_ can achieve the maximum exposure of the catalytically active interface through the dual mechanism of preferential removal of the C/N component in Ni–Fe–C–N by chemical oxidation and enhancement of the plasma etching kinetics, which exhibits the reconfiguration of the Prussian blue analogues with oriented structures.

In Fig. S2, the N_2_ adsorption–desorption isotherm recorded at 77 K displays a characteristic type-IV profile with a clear hysteresis loop in the *P*/*P*_0_ range of 0.8–1.0, confirming the presence of abundant mesopores. A rapid uptake at very low relative pressures (*P*/*P*_0_ < 0.1) indicates additional microporosity, while the steep rise near saturation pressure points to macropores or inter-particle voids. BJH analysis further reveals a trimodal pore-size distribution centered at 1 nm, 4–6 nm, and 50–120 nm, corroborating the coexistence of micro-, meso- and macropores. Such a hierarchical architecture, together with a BET surface area of 43.4 m^2^ g^−1^, provides plentiful accessible interfaces and short diffusion paths for electrolyte penetration and gas transport.

**Fig. 3:**
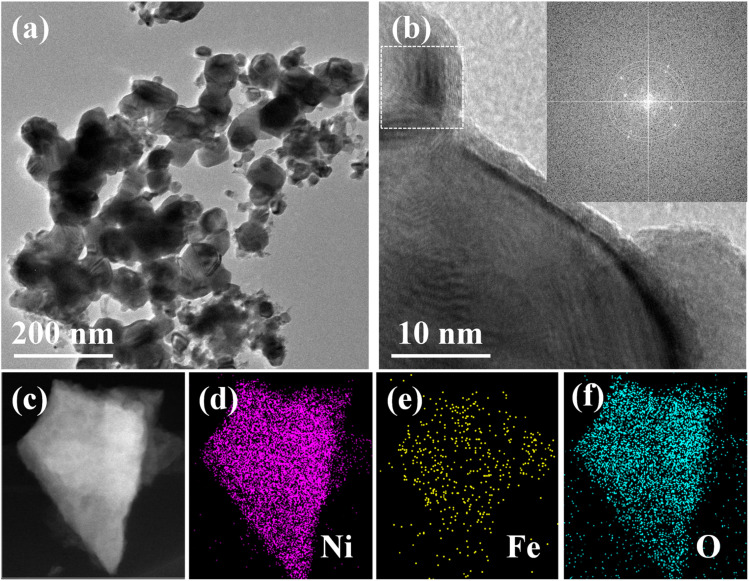
TEM (a), HRTEM (b), HAADF image (c) and corresponding elemental mapping (d–f) of NiFe PBA-Ar/O_2_.

The NiFe PBA-Ar/O_2_ sample was detached from the nickel foam *via* high-power ultrasonication and subjected to TEM characterization. As shown in Fig. 3a, the plasma-treated sample exhibits nanoparticle-like morphology. High-resolution TEM (HRTEM) analysis of a selected particle (Fig. 3b) reveals distinct lattice fringes in the boxed region. The corresponding fast Fourier transform (FFT) pattern (inset in the upper right corner) displays three diffraction rings, which are indexed to the (220), (331), and (422) crystallographic planes of spinel-type NiFe_2_O_4_, with interplanar spacings of 0.294 nm, 1.91 nm, and 1.70 nm, respectively. These results confirm that the O_2_ plasma chemically reacts with the pristine PBA, leading to the formation of nickel–iron oxides. Furthermore, the energy-dispersive X-ray spectroscopy (EDS) elemental mapping (Fig. 3c–f) demonstrates the homogeneous distribution of Ni, Fe, and O within the same particle.

**Fig. 4:**
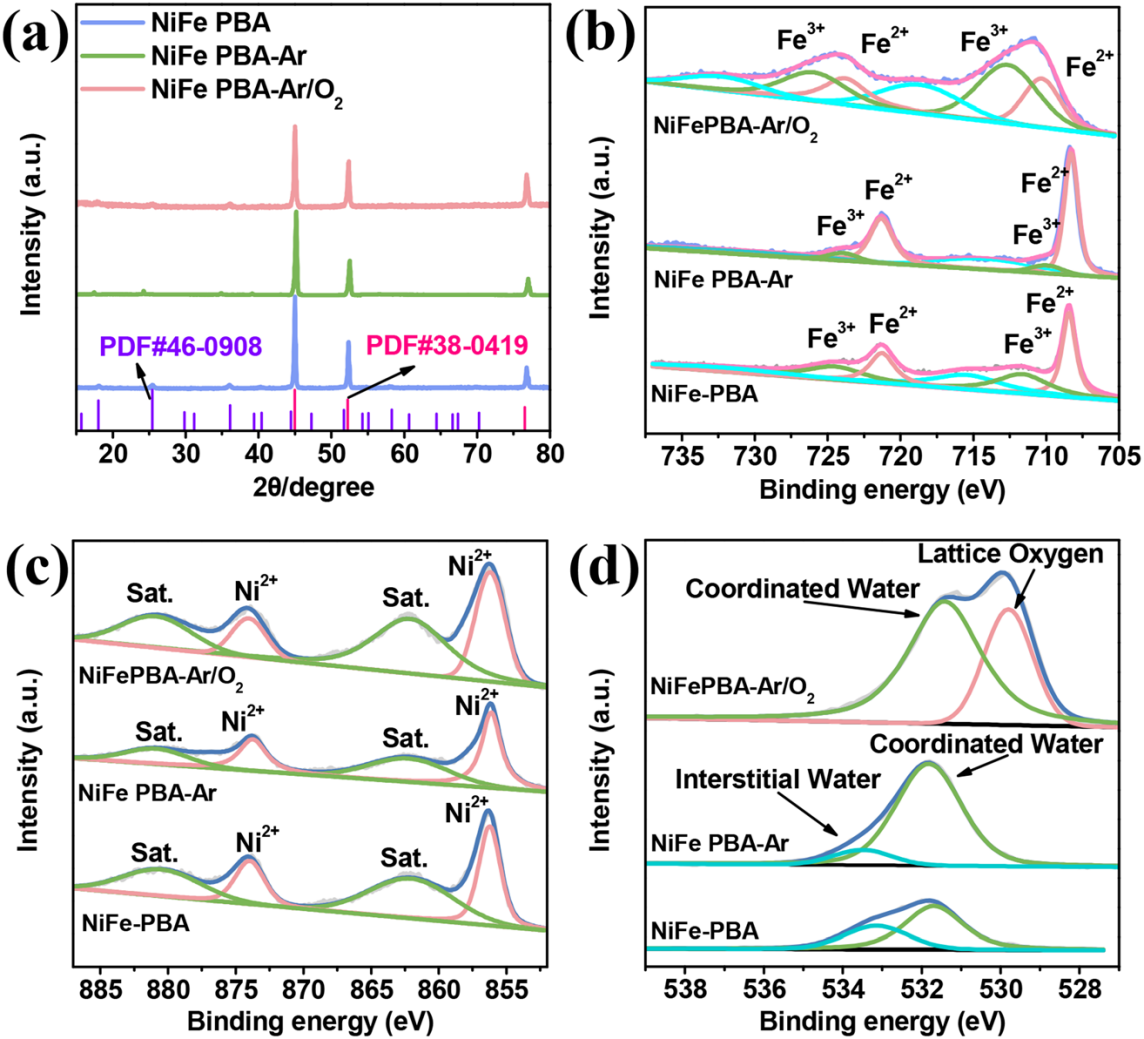
XRD patterns of NiFe PBA, NiFe PBA-Ar and NiFe PBA-Ar/O_2_ (a); fine spectra of Fe 2p (b), Ni 2p (c), and O 1s (d) XPS in NiFe PBA, NiFe PBA-Ar, and NiFe PBA-Ar/O_2_ catalysts.

X-ray diffraction (XRD) technique was employed to characterize the phase evolution of the PBA samples before and after plasma etching. As shown in Fig. 4a, the XRD pattern of the NiFe PBA sample exhibits distinct diffraction peaks of metallic nickel at 44.9°, 52.2°, and 76.5°, corresponding to the substrate signals from the nickel foam. Meanwhile, the characteristic diffraction peaks of PBA located at 25.4°, 18.0°, 36.1°, and 58.3° confirm the successful attachment of NiFe PBA onto the nickel foam. After plasma etching (whether it is pure argon plasma or a plasma of a mixed gas of argon and oxygen), the position of diffraction peaks remains nearly unchanged. However, the intensity of PBA diffraction peaks decreases due to plasma bombardment-induced lattice defects (causing lattice expansion and localized atomic disordering, thereby reducing crystallinity). In the samples treated with argon–oxygen mixed gas plasma, the intensity of the diffraction peaks decreased more significantly, as the oxygen plasma not only induces physical bombardment but also triggers chemical reactions. These modifications are favorable for promoting electron transfer in electrocatalytic processes. Nevertheless, no new diffraction peaks attributable to phases produced by the plasma bombardment are detectable in the XRD patterns. This may be because the plasma-affected layer is extremely shallow, lying beyond the detection limit of XRD.

The XPS technique, highly sensitive to surface chemical environments, effectively reveals the plasma-induced chemical modifications on the PBA surface. As shown in Fig. S2 and Table S1, the XPS survey spectra reveals that O_2_ plasma treatment significantly reduces the elemental content of C and N. This occurs because nonmetallic elements in the PBA framework react with energetic reactive O_2_ plasma to form nitrogen oxides and carbon oxides, which are subsequently removed by the vacuum system, while metallic elements are converted into corresponding oxides. As illustrated in Fig. 4b, the Fe 2p high resolution spectra of the NiFe PBA sample exhibit characteristic peaks of low-spin Fe^2+^ (708.44 eV) and high-spin Fe^3+^ (711.61 eV). NiFe PBA adopt a face-centered cubic (fcc) framework where Fe ions occupy two distinct sites, Fe^3+^ coordinates with nitrogen (Fe^3+^–N≡C) in the [Fe(CN)_6_]^3−^ units, While Fe^2+^ coordinates with carbon (Fe^2+^–C≡N) in the [Ni(CN)_4_]^2−^ or similar units. This dual-site configuration inherently stabilizes mixed valency due to charge balance requirements within the lattice.^31^

After argon plasma treatment, the binding energy of Fe^2+^ (708.4) slightly decreases, likely resulting from C and N defects generated by plasma bombardment. Simultaneously, the NiFe PBA-Ar sample exhibits characteristic peaks of Fe^3+^ (710.1 eV) with extremely low content, indicating that Ar plasma bombardment may correspondingly induce reduction reaction.^32^ In contrast, the reactive Ar/O_2_ plasma treatment induces a distinct high-spin state in the NiFe PBA-Ar/O_2_ sample: the Fe^2+^ peak shifts positively to 710.3 eV, accompanied by a new Fe^3+^ peak at 712.8 eV. This indicates that reactive O_2_ plasma interacts with C and N elements in the PBA framework, forming carbon oxides and nitrogen oxides that are removed from the lattice, while metallic Fe is retained as oxidized species. The presence of high-valent Fe (Fe^3+^) optimizes the adsorption/desorption of intermediates during the oxygen evolution reaction (OER), enhancing electrocatalytic efficiency through tailored electronic structure and reduced energy barriers.

For the Ni 2p fine spectra (Fig. 4c), the characteristic Ni^2+^ peaks in the Ni 2p_3/2_ region remain nearly unchanged at 856.3 eV across all samples,^33^ consistent with the high-spin state of Ni coordinated to nitrogen atoms in the cyanide ligands within the PBA framework. The O 1s fine spectra (Fig. 4d) further reflects the chemical modifications induced by reactive plasma. In the pristine NiFe PBA sample, two oxygen-related peaks are observed at 531.7 eV and 533.1 eV, corresponding to lattice water (H_2_O molecules directly coordinated to metal ions) and interstitial water (H_2_O trapped in lattice pores/defects without coordination), respectively. The Ar plasma-treated sample shows no new oxygen species, confirming its limited physical interaction with PBA. In contrast, the Ar/O_2_ plasma-etched sample exhibits a distinct lattice oxygen peak at 529.8 eV, attributed to the formation of Ni/Fe oxides *via* oxidative reactions.^34^

To verify the synergistic effect of the Ar and O_2_ plasma, the NiFe PBA-O_2_ sample was also characterized by XPS, SEM and TEM. As depicted in Fig. S3a, the vigorous etching induced by the O_2_-rich plasma almost completely destroys the original PBA framework, resulting in a fragmented, highly porous architecture. The inset of Fig. S3a displays the corresponding TEM image, where numerous nano-sized pores can be clearly distinguished. These voids stem from the removal of the carbon- and nitrogen-based ligands that were selectively etched away by the oxidative plasma.

Fig. S3b–d display the high-resolution XPS spectra of Fe 2p, Ni 2p, and O 1s for the NiFe PBA-O_2_ sample. Relative to the NiFe PBA-Ar/O_2_ sample, etching with pure O_2_ plasma shifts the iron peaks to higher binding energies. In the Fe 2p_3/2_ component, the Fe^2+^ characteristic peak now appears at 711.4 eV, while the Fe^3+^ peak is further shifted to 714.0 eV, confirming the stronger oxidative effect of the O_2_ plasma. In the Ni 2p_3/2_ spin–orbit split peak, the characteristic peak of Ni^2+^ remains at 856.3 eV. The high-resolution O 1s spectrum shows that lattice oxygen is dominant, while the proportion of coordinated water in the PBA structure decreases, indicating that the fundamental PBA framework has been damaged by the O_2_ plasma. In summary, introducing a moderate amount of high-valent Fe can markedly enhance OER kinetics by tuning the Ni–O–Fe electronic structure; however, excessive Fe content or valence dilutes Ni active sites, lowers conductivity, and accelerates structural degradation, thereby reducing both activity and stability.

Combined XRD, XPS, and SEM analyses reveal that Ar plasma primarily induces physical interactions (*e.g.*, defect generation and crystallite refinement) without altering PBA's chemical composition. In contrast, reactive O_2_ plasma triggers chemical reactions with C/N elements, producing gaseous byproducts (*e.g.*, CO_*x*_, NO_*x*_) that are evacuated by the vacuum system, while metallic species remain as high-valent oxides. This process simultaneously generates abundant porosity in the PBA structure. These synergistic modifications—chemical environment optimization (enhanced high-valent metal species) and morphological restructuring (porous frameworks)—collectively improve charge/proton transfer kinetics during the oxygen evolution reaction (OER).

Significant surface changes *via* XPS but minimal structural changes in XRD were observed. It originates from fundamental differences in probe depth between the techniques: plasma processing is intrinsically surface-limited (<10 nm), aligning precisely with XPS's extreme surface sensitivity (2–10 nm). This shallow modification zone primarily affects chemical states detectable by XPS (*e.g.*, oxidation-induced binding energy shifts) while remaining undetectable to XRD due to its bulk-probing nature (∼μm depth) and reliance on long-range crystal periodicity. Consequently, XRD patterns – reflecting preserved bulk crystallinity – exhibit minimal change, as the altered surface layer lacks structural coherence or sufficient thickness to generate measurable Bragg diffraction.

**Fig. 5:**
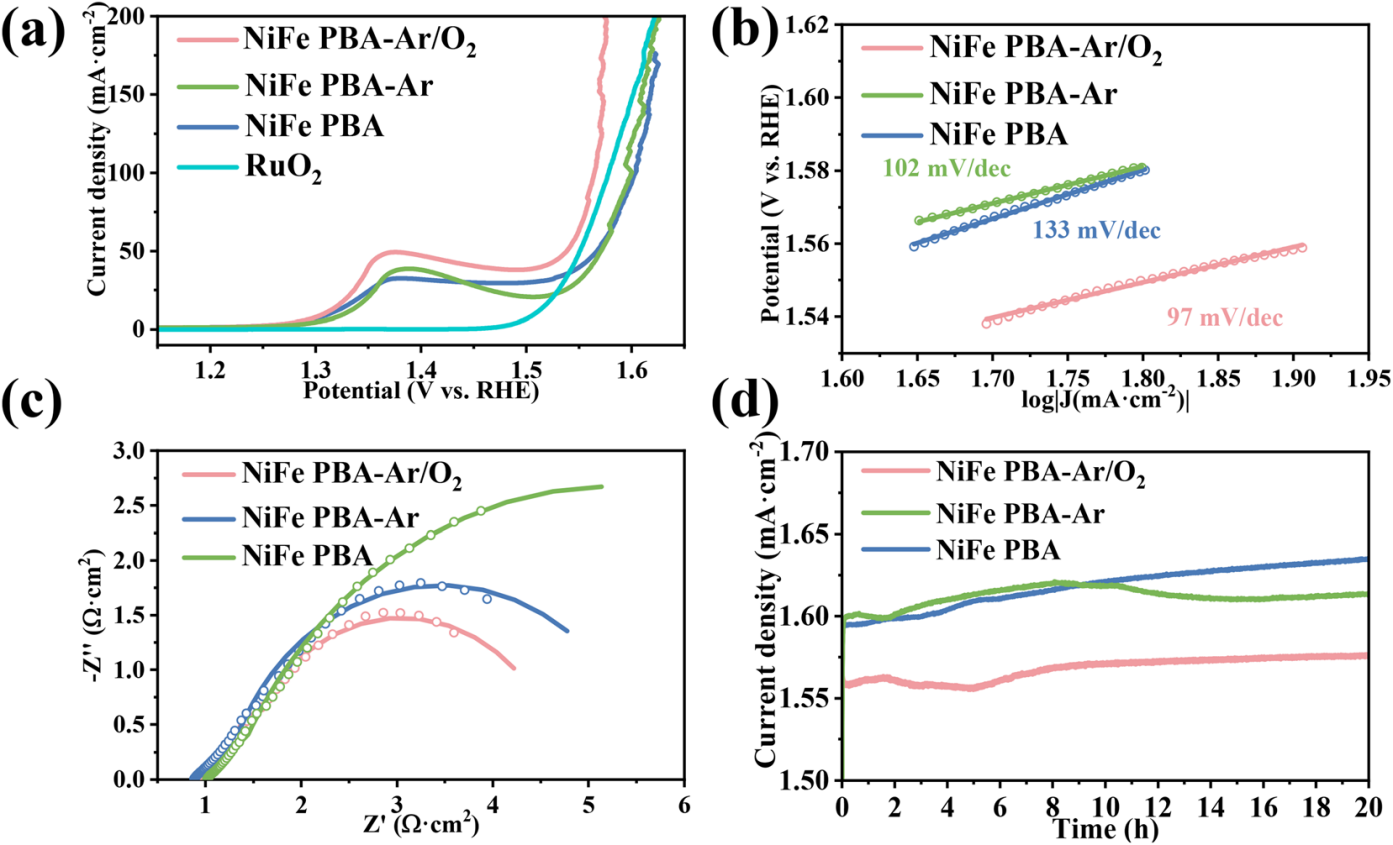
LSV curves of NiFe PBA, NiFe PBA-Ar and NiFe PBA-Ar/O_2_ catalysts in 1 M KOH solution (a); Tafel slope (b); EIS spectrum (c) and stability test curve (d).

The oxygen evolution reaction (OER) performance of the three samples was compared with that of the commercial RuO_2_ catalyst in 1.0 M KOH. To exclude the catalytic contribution of the nickel foam itself, the plain nickel foam current collector was also subjected to LSV testing. The results, as shown in Fig. S5, indicate that its catalytic performance is suboptimal. As shown in the LSV polarization curves (Fig. 5a), the NiFe PBA-Ar/O_2_, NiFe PBA-Ar, and NiFe PBA samples exhibited distinct oxidation currents within the potential window of 1.3–1.5 V (*vs.* RHE), indicating catalyst surface reconstruction. The distinct anodic peaks in NiFe PBA-Ar/O_2_ NiFe PBA-Ar, and NiFe PBA samples arise from the pre-oxidation of Ni^2+^ to Ni^3+^, generating catalytically active NiOOH species essential for OER initiation. Plasma etching (Ar/O_2_) intensifies this peak by removing cyanide ligands and exposing metallic sites. To validate the surface reconstruction, *in situ* electrochemical Raman spectroscopy was performed on the NiFe PBA-Ar/O_2_ sample. As depicted in Fig. S3, no characteristic Raman peaks were observed at 1.25 V. However, upon increasing the potential to 1.30–1.40 V, a Raman scattering peak emerged at 525.2 cm^−1^, attributed to the Ni–O vibration in disordered NiOOH. At higher potentials (1.40–1.45 V), two additional peaks at 480 and 563 cm^−1^ were detected, confirming the phase transition from disordered NiOOH to ordered γ-NiOOH. Specifically, the peak at 480 cm^−1^ corresponds to the bending vibration of Ni^3+^–O (E_g_ mode), while the peak at 563 cm^−1^ is assigned to the stretching vibration of Ni^3+^–O (A_1g_ mode), reflecting enhanced symmetry of lattice oxygen. Notably, a broad peak in the range of 900–1200 cm^−1^ appeared at potentials above 1.45 V, which is associated with M–OOH and *OOH. Plasma treatment with mixed gases (Ar/O_2_) induces more intensive structural reconstruction of catalysts prior to OER catalysis, forming a hydroxide oxide layer (NiOOH, FeOOH) that favors the Adsorbate Evolution Mechanism (AEM). Furthermore, the LSV curves of plasma-treated samples under different power conditions (Fig. S4) demonstrate that power adjustments modulated the surface chemical states, thereby influencing catalytic performance.

The Tafel slopes derived from the LSV curves are shown in Fig. 5b. The NiFe PBA-Ar/O_2_, NiFe PBA-Ar, and NiFe PBA samples exhibited Tafel slopes of 97, 102, and 133 mV dec^−1^, respectively, indicating that the Ar/O_2_ plasma-treated sample possesses optimal OER kinetics. The lower Tafel slope (97 mV dec^−1^) of NiFe PBA-Ar/O_2_ suggests faster current density enhancement under identical overpotentials, which is closely related to its higher intrinsic activity and more efficient charge transfer processes.^35^ The electrochemical impedance spectra (EIS) of the three samples (Fig. 5c) revealed charge transfer resistances (*R*_ct_) of 3.9, 4.6, and 10.2 Ω cm^2^ for NiFe PBA-Ar/O_2_, NiFe PBA-Ar, and NiFe PBA, respectively, further confirming the significantly reduced charge transfer barrier at the NiFe PBA-Ar/O_2_ catalyst interface. Combined with the aforementioned physical characterization results, it can be concluded that the hybrid plasma treatment promotes the formation of a nickel–iron metal oxide layer enriched with high-valent Fe species, which facilitates the OER proceeding, thereby enhancing reaction kinetics.

The chronopotentiometric polarization curves at a constant current density of 100 mA cm^−2^ are shown in Fig. 5d. The NiFe PBA-Ar/O_2_ sample demonstrated exceptional stability, with a minimal potential decay of only 40 mV over 20 hours, outperforming both NiFe PBA-Ar and NiFe PBA. A comparative analysis with previously reported catalysts (Table S2) highlights that the Ar/O_2_ plasma treatment is a highly effective surface modification strategy for significantly boosting catalytic efficiency.

Post-stability XPS analysis (Fig. S8a–c) reveals significant surface reconstruction: Fe 2p spectra show retained Fe^3+^ with intensified satellite peaks (712.9 eV and 716.5 eV), indicating hydroxyl coordination and high-spin state persistence despite partial leaching. Ni 2p exhibits a 0.9 eV positive shift (857.2 eV), confirming Ni^3+^-rich γ-NiOOH formation. In Fig. S8c, O 1s spectra dominance by hydroxyl species (532.1 eV) and reduced lattice oxygen signal (530.1 eV) demonstrate transition toward a hydroxylated interface favorable for the lattice oxygen oxidation pathway. These changes collectively enhance intermediate adsorption energetics. In Fig. S8d, SEM images of post-stability samples reveal agglomerated spherical particles on the catalyst surface. Under high oxidative potential, prolonged exposure, and vigorous bubbling, the structure of the catalyst has collapsed, resulting in a reconstruction of the catalyst's surface. The hierarchical porous structure obtained after plasma etching has been partially preserved, thereby ensuring continued mass transfer under high current density operation.

## Conclusion

In summary, the synergistic Ar/O_2_ plasma engineering strategy successfully enhances the OER performance of NiFe Prussian blue analogues by synergizing combining the physical bombardment of Ar in the plasma with the chemical oxidation effect of the O_2_ plasma. The hybrid plasma treatment creates porosity through selective ligand removal, exposes catalytically active interfaces, and stabilizes high-spin Fe^3+^ species *via* electronic modulation, thereby optimizing intermediate adsorption/desorption energetics. The resultant NiFe PBA-Ar/O_2_ catalyst achieves a low overpotential of 334 mV at 100 mA cm^−2^, a reduced Tafel slope of 97 mV dec^−1^, and exceptional stability (40 mV decay over 20 h), outperforming noble-metal benchmarks. Mechanistic studies reveal that plasma-induced defects and high-valent metal centers facilitate adsorbate evolution mechanism (AEM), accelerating reaction kinetics. This work demonstrates the efficacy of non-equilibrium plasma technology in reconciling structural integrity with catalytic activity for MOF-based materials, offering a scalable and cost-effective route to advance green hydrogen production through water electrolysis. Future efforts will focus on extending this strategy to diverse MOF architectures to unlock broader applications in sustainable energy systems.

## Conflicts of interest

There are no conflicts to declare.

## Supplementary Material

RA-015-D5RA03979G-s001

## Data Availability

Further data are available from the corresponding author upon reasonable request. All data supporting the findings of this study are available within the article and its supplementary information files (SI). Supplementary information is available. See DOI: https://doi.org/10.1039/d5ra03979g.
