# Supplementary material for: High-efficiency oxygen evolution electrocatalysis enabled by Ar/O2 plasma-induced synergistic modifications in NiFe Prussian blue analogue systems
Source: RSC Adv. 2025 Sep 30;15(43):36206–12. doi: 10.1039/d5ra03979g (PMC12481245; doi:10.1039/d5ra03979g)
Supplement: RA-015-D5RA03979G-s001 [file RA-015-D5RA03979G-s001.pdf]

Supplementary Information (SI) for RSC Advances.
This journal is © The Royal Society of Chemistry 2025

High-Efficiency Oxygen Evolution Electrocatalysis Enabled by Ar/O_2 Plasma-Induced Synergistic Modifications in NiFe Prussian Blue Analogue Systems

Yaoyao He[a], Jin Fang[a], Wenyuan Zhang[a], Nnditshedzeni Eric Maluta[b], Fhulufhelo Nemangwele[a,b], Li Zhang[a], Hui Lv[a,c], Pei Hu[a] and Zhuo Peng[a]*

[a] China-South Africa PV-Hydrogen Energy Joint Research Center, School of Science, Hubei University of Technology (HBUT), Wuhan 430068, China.

[b] Department of Physics, University of Venda, Thohoyandou 0950, South Africa.

[c] School of Physics and Electromechanical Engineering, Hubei University of Education, Wuhan 430068, China.

Experimental Section

All chemical reagents were purchased from Sinopharm Group and used directly without purification. The nickel foam is cut into 2cm*3cm size and the surface is rinsed with anhydrous ethanol to rinse oil and other impurities. Next, the nickel foam was immersed in a 1M HCl solution for 10 minutes to remove nickel oxide impurities from the surface. The nickel foam was then rinsed sequentially using deionized water and anhydrous ethanol to ensure that residual HCl was thoroughly removed. Finally, the treated nickel foam was stored in anhydrous ethanol for subsequent use.

Synthesis of the catalysts

Add 0.01 mol ($K_3[Fe(CN)_6]$) potassium ferricyanide to 50 mL of hydrochloric acid solution with a concentration of 0.2 M and magnetically stir at room temperature until complete dissolution to form a clarified solution. The pretreated nickel foam was immersed into the above solution and allowed to stand at room temperature for 12 h. The nickel foam was then taken off and washed several times with ultrapure water and anhydrous ethanol to remove the unreacted residues on the surface, and placed in an oven to dry overnight, and the resulting product was labeled as NiFe PBA.

The processed nickel foam is placed into the reactive ion etching machine (Tailong Electronics, RIE-150). When the vacuum reaches 10^{-4} Torr, gases are introduced to commence the etching experiment. The argon gas flow rate is 100 cc, the oxygen gas flow rate is 10 cc, and the power is set to 90 W. The front and back sides were treated for five minutes, and the MOF catalyst material containing Ni-Fe oxides was obtained after the etching was completed, which is labeled herein as NiFe PBA-Ar/O_2. The labeling methods of other samples were differentiated by the differences in process treatments. In addition, the sample treated only by argon is labeled as NiFe PBA-Ar; the sample treated only by oxygen is labeled as NiFe PBA-O_2 (oxygen gas flow rate is 100 cc).

The loading amounts of the various catalysts were calculated using the weighing method and were determined to be 1.67 mg/cm^2 (NiFe PBA), 1.53 mg/cm^2 (NiFe PBA-Ar), 1.48 mg/cm^2 (NiFe PBA-Ar/O_2), and 1.65 mg/cm^2 (NiFe PBA-O_2), respectively.

Material characterization

The microstructures of all samples were characterized via Field-emission scanning electron microscopy (FESEM, Nova Nano SEM 450) and transmission electron microscopy (TEM, Tecnai G2 F30). X-ray diffraction (XRD, X'Pert PRO diffractometer) was employed to investigate the crystallographic structure with Cu Kα radiation (λ=1.5418 Å). The surface chemical environment was tested by X-ray photoelectron spectroscopy (XPS, AXIS-ULTRA DLD-600W). The in-situ Raman spectra was analyzed by a confocal Raman microscope (Horiba XploRA plus).

Electrochemical measurement

The electrodes were fully characterized using a CorrTest electrochemical workstation, covering a variety of techniques such as linear scanning voltammetry (LSV), Tafel curve analysis, electrochemical impedance spectroscopy (EIS), and timed current testing. All experiments were based on a three-electrode system in an alkaline environment (1 M KOH), where the foam nickel catalyst is cut into 2*1 cm^2 pieces, clamped with an electrode holder, exposing only a 1*1 cm^2 area to serve as the working electrode. and the reference electrode

was Hg/HgO. During the experiments, the potential of the working electrode needed to be converted to reversible hydrogen electrode potential (RHE), which was calculated as follows:

$$RHE = E_{(SCE)} + 0.0592 \times \mathrm{pH} + 0.098 \quad (1)$$

Linear Scanning Voltammetry (LSV) is used to obtain a linear scanning voltammetric curve by applying a potential with a constant rate of change to the working electrode and measuring its effect on the current density. Specific parameters include: OER test range of 1.1 to 1.8 V and a scan rate of 5 mV/s. The slope of the Tafel curve reflects the catalyst activity and the curve is able to respond to the relationship between the steady-state current density and overpotential, and the Tafel equation is expressed as:

$$\eta = \mathrm{a} + \mathrm{b} \times \log|\mathrm{j}| \quad (2)$$

where η denotes the overpotential, a and b are Fennel's constants, and j represents the current density. The linear region was selected within the kinetically controlled regime of 1.54-1.60 V vs. RHE, which corresponds to an overpotential (η) range of 310–370 mV (OER onset potential: ~1.23 V vs. RHE). This window avoids: low-potential non-faradaic currents and high-potential diffusion limitations. The AC amplitude for the EIS test was set to 5 mV with a frequency range from 100 kHz to 0.01 Hz. The voltage-to-current ratio of the AC signals was measured by applying a series of small amplitude AC signals of different frequencies to the system. EIS was fitted using Zview software to obtain solution resistance (R_s) and charge transfer resistance (R_{ct}) through a single-capacitance equivalent circuit. All polarization curves were calibrated by IR drop compensation method to eliminate the effect of solution resistance R_s. The stability of the catalyst is tested using a constant current protocol.

In situ Raman spectra tests: A photograph of in situ Raman setup was shown in Figure. S1. An electrolytic cell with three electrode configuration was used for the electrochemical test. A 15*15 mm sample was used as working electrode, a Pt wire and Ag/AgCl (saturated KCl) electrodes were used as counter and reference electrode, respectively. During Raman test, 1 M KOH solution was continuously fed into the electrolytic cell at a rate of 10 ml/min. Raman spectra was collected in the range of 200 to 2000 cm^{-1} using a 638-nm laser.

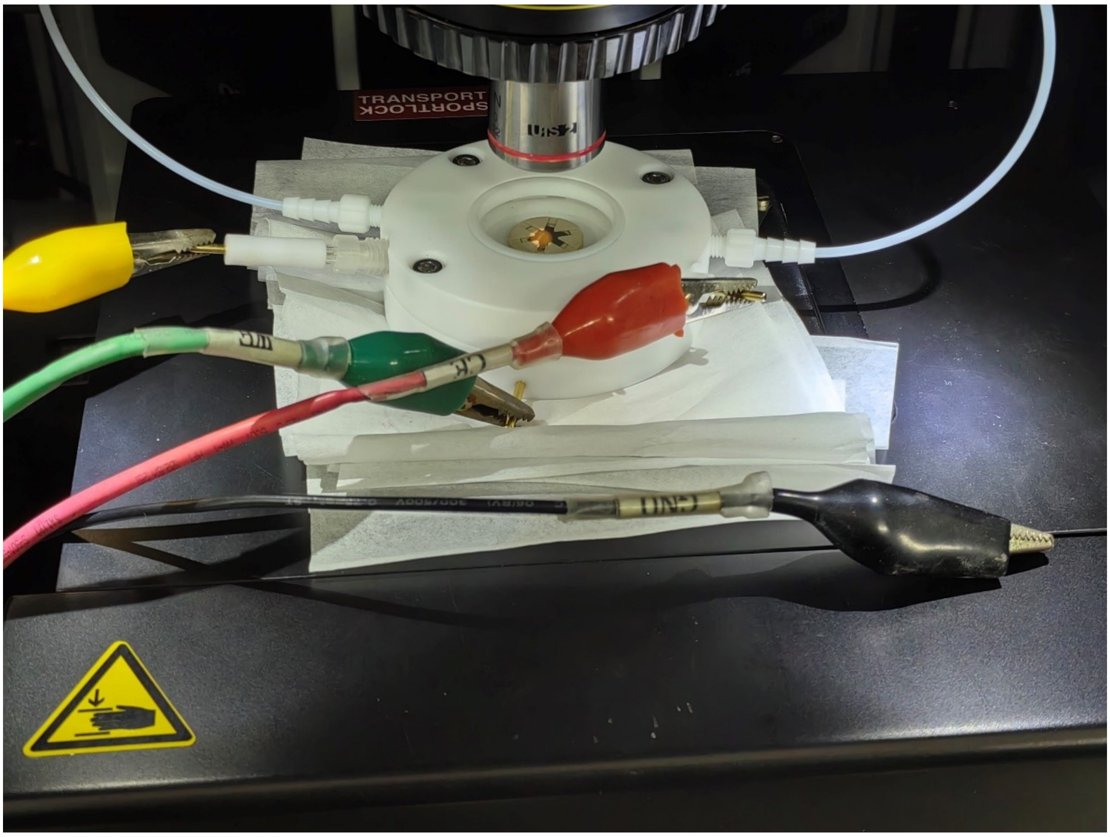

Figure S1. Photo of the electrochemical in situ Raman testing cell

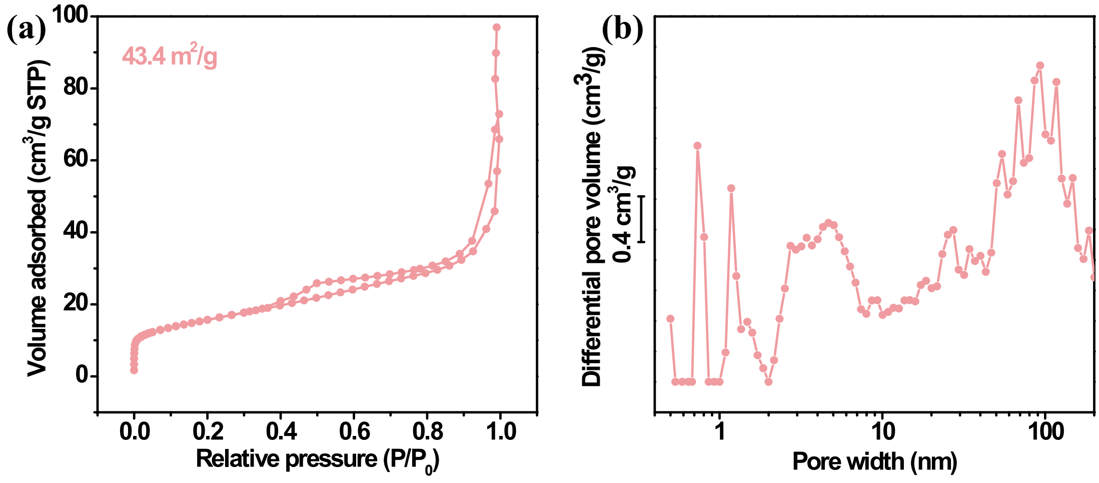

Figure S2. (a) N_2 isothermal adsorption curve (at 77 K) and BJH pore size distribution

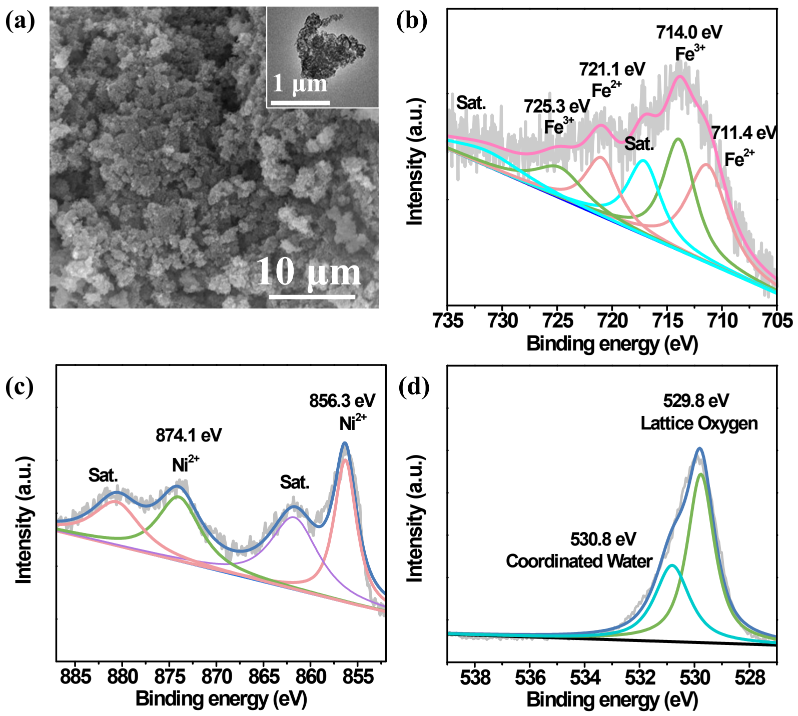

Figure S3. SEM and TEM(the upper right corner) image of NiFe PBA-O_2 (a);fine spectra of Fe 2p (b), Ni 2p (c), and O 1s (d) XPS in NiFe PBA-O_2

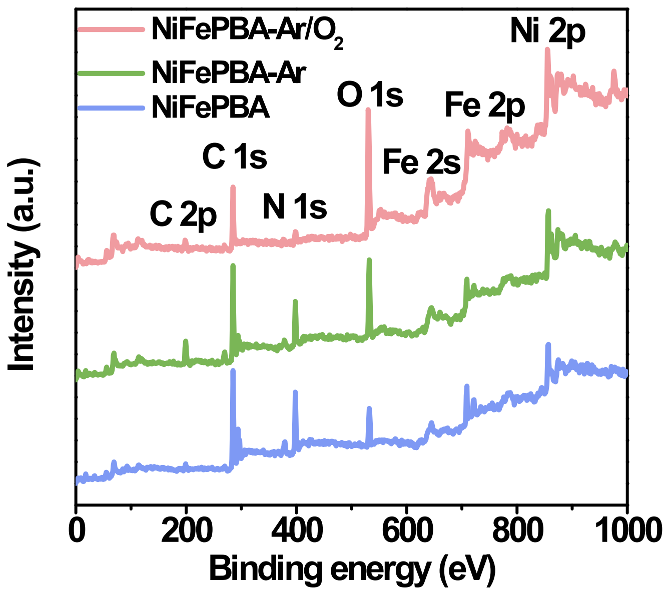

	NiFePBA- Ar/O2	NiFePBA- Ar	NiFePBA
C	34.32	56.35	63.81
Fe	10.26	4.1	4.17
N	5.64	13.63	17.04
Ni	13	8.91	5.57
O	36.77	17.02	9.41

Figure S4. XPS survey spectra of NiFe PBA-Ar and NiFe PBA-Ar/O_2

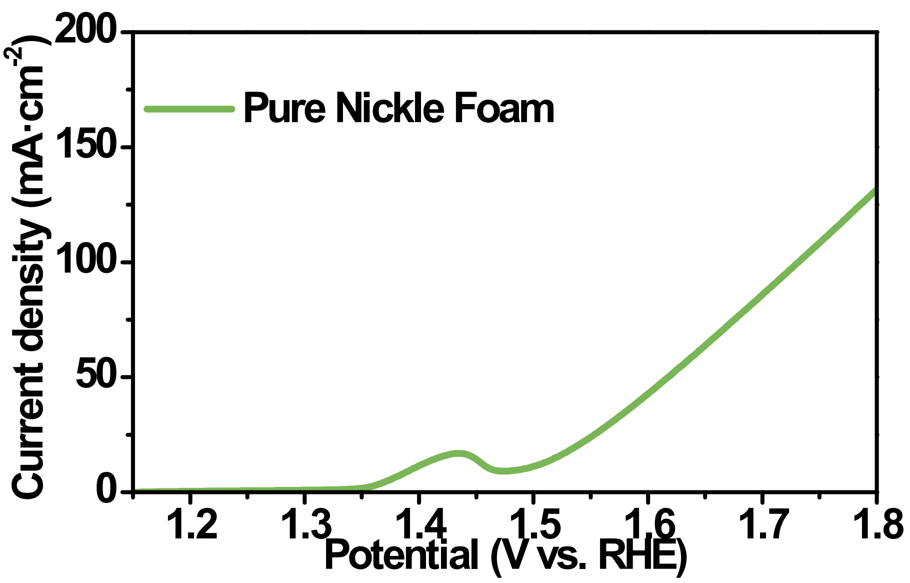

Figure S5.LSV curves of pure nickle foam

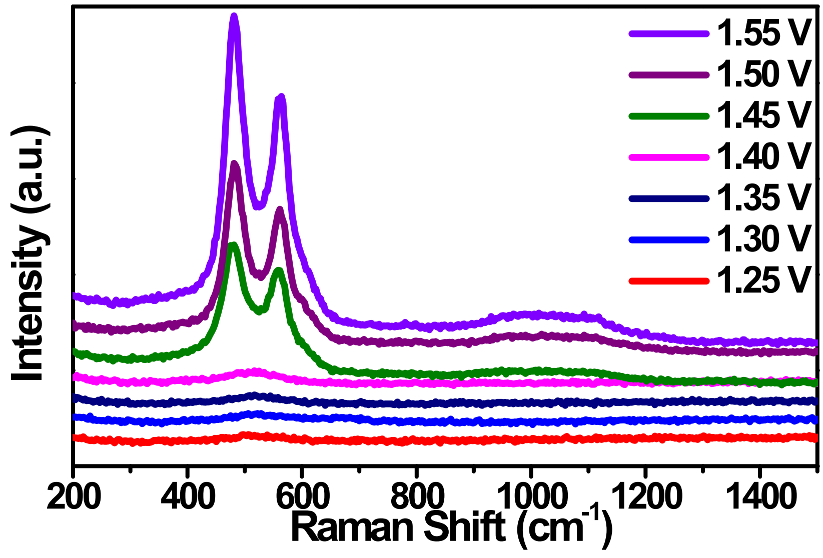

Figure S6. In-situ Raman Spectroscopy Curves of NiFe PBA-Ar/O_2 during Electrochemical Process

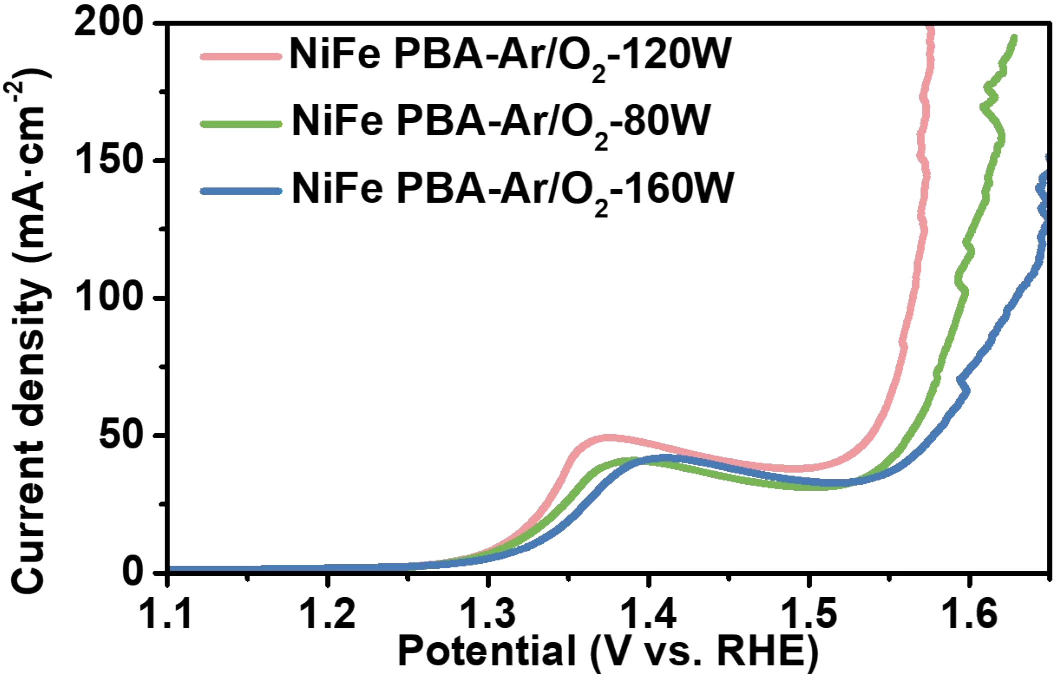

Figure S7. LSV curves in 1.0 M KOH solution of NiFe PBA-Ar and NiFe PBA-Ar/O_2

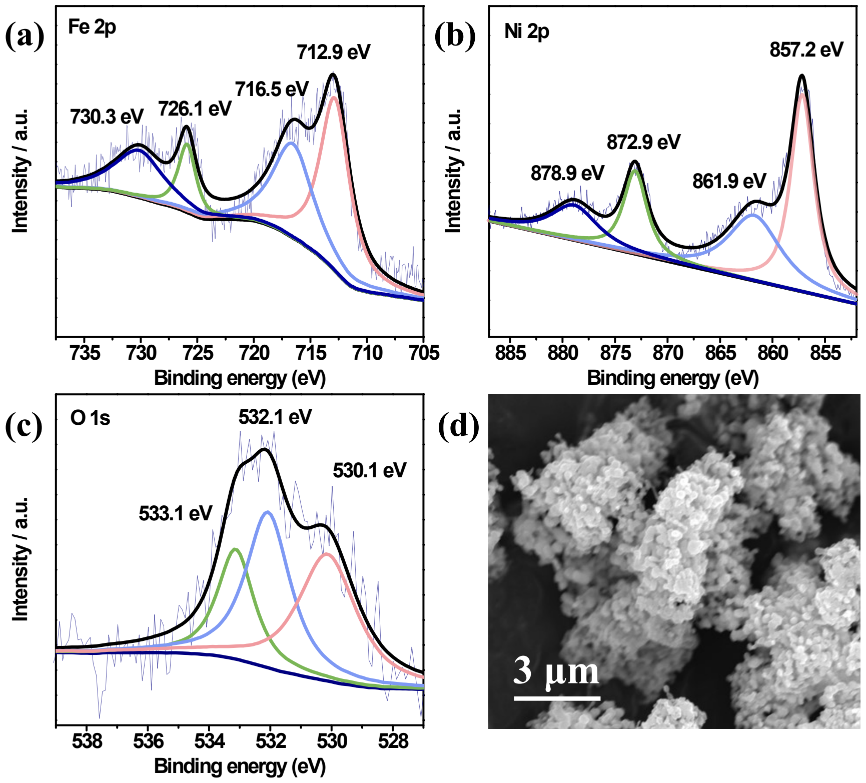

Figure S8. Post-stability XPS analysis(a)-(c) of NiFe PBA-Ar/O_2; Post-stability SEM image of NiFe PBA-Ar/O_2

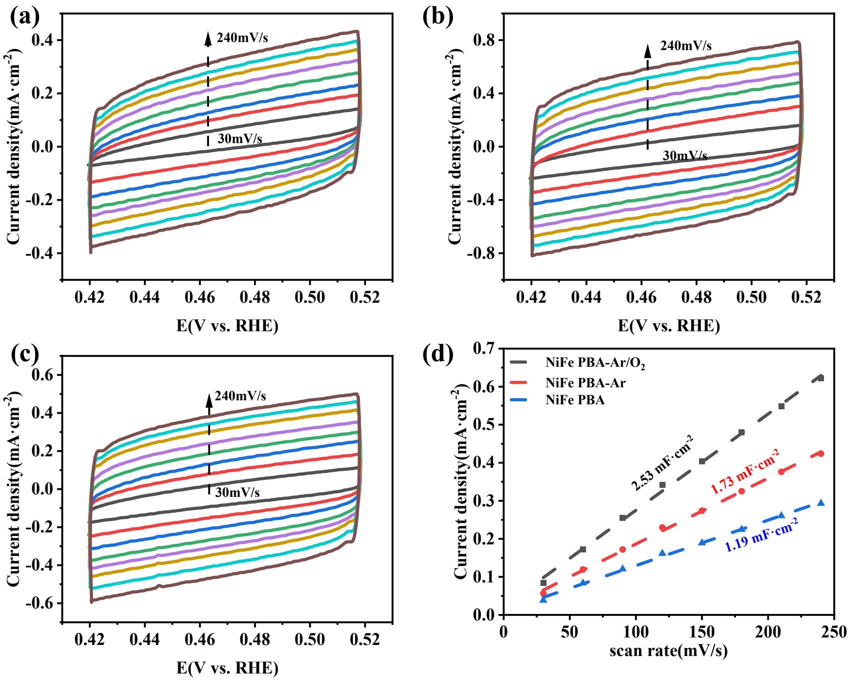

Figure S9.CV curves of NiFe PBA (a), NiFe PBA-Ar/O_2 (b) and NiFe PBA-Ar (c);C_{dl} of samples (d)

Table S1. Atomic Ratio of Elements Obtained from XPS Analysis

	NiFe PBA-Ar/O_2	NiFe PBA-Ar	NiFe PBA
C	34.32	56.35	63.81
Fe	10.26	4.1	4.17
N	5.64	13.63	17.04
Ni	13	8.91	5.57
O	36.77	17.02	9.41

Table S2. Comparison of Oxygen Evolution Electrocatalytic Activity between NiFe-based Catalysts in Literature and Catalysts in This Work

	Environment	j/(mA cm^{-2})	η / mV	Refs
$Ni_{1-x}Fe_xOOH$-LDH	1 M KOH	100	410	*Materials Today Advances*.2025, 25, 100560

A-NiFeV/NF	1 M KOH	100	313	Journal of Colloid and Interface Science, 2024, 653, 721-729
Fe-MoVNi	1 M KOH	100	374	*Molecular Catalysis,* 2023, 542, 113132
NiFe@C 5:3	1 M KOH	100	438	*International Journal of Hydrogen Energy,* 2022, 47, 18955-18962
NiFe LDH 4:1	1 M KOH	100	332	*Materials Today Sustainability,* 2025, 31, 101130
$NiFeF_5$	1 M KOH	100	349	*ChemPhysChem,* 2025, 26, e202400701.
HP Ni-P	1 M KOH	100	331	*ECS Meeting Abstracts.* 2020, 1, 1583.
Fe-N-C/ $Ni_{0.75}Fe_{0.25}$	1 M KOH	100	312	*Electrochimica Acta,.* 2018, 281, 684-691.
$Ni_{0.1}Co_{0.9}$-MOF	1 M KOH	100	384	*Journal of Alloys and Compounds,* 2023, 947 169498.
Ni–Mn–Co–Fe	1 M KOH	100	447	*Renewable Energy.* 2024, 237, 121688.
NiMnFe-ex	1 M KOH	100	321	*Electrocatalysis.* 2025, 1868-5994
Ni-Mo-Fe	1 M KOH	100	408	*International Journal of Hydrogen Energy.* 2021, 46, 3821-3822.
$V_{0.03}$Fe- $Ni_3S_2/Ni(OH)_2$	1 M KOH	100	385	*Surfaces and Interfaces.* 2025, 58, 105864.
N-$NiFeSe_2$	0.5 M H_2SO_4	100	332	*Electrochem. Commun.* 2021, 131, 107118.
$NiFeO_xH_y$-HS	1 M KOH	100	305	*J. Mater. Sci.* 2020, 55, 15140.
Fe2Ni5-T175	1 M KOH	20	262	*Fuel 400 (2025) 135730*
MNFO-V_O	1 M KOH	100	390	*International Journal of Hydrogen Energy 137*

				(2025) 73–82
$Nd_{0.1}Ni_{0.9}Co_2O_4$	1 M KOH	100	520	*Journal of Colloid and Interface Science 691 (2025) 137411*
Co-NiFe2O4/NiTe2	1 M KOH	10	225	*Materials Today Chemistry 47 (2025) 102840*
NiFeP	1 M KOH	10	210	*Journal of the American Chemical Society. 2024, 146, 12548-12556*
NiFe-LDH/ $NiFe_2O_4$/ NiFe-LDH	1 M KOH	100	390	*Nano Research. 2024, 17, 6646*
**NiFe PBA-Ar/O_2**	**1 M KOH**	**100**	**334**	**This work**
